# Susceptibility and characteristics of infections in patients with glucocorticoid excess or insufficiency: the ICARO tool

**DOI:** 10.1530/EJE-22-0454

**Published:** 2022-09-14

**Authors:** Marianna Minnetti, Valeria Hasenmajer, Emilia Sbardella, Francesco Angelini, Chiara Simeoli, Nicola Di Paola, Alessia Cozzolino, Claudia Pivonello, Dario De Alcubierre, Sabrina Chiloiro, Roberto Baldelli, Laura De Marinis, Rosario Pivonello, Riccardo Pofi, Andrea M Isidori

**Affiliations:** 1 Department of Experimental Medicine, Sapienza University of Rome – Policlinico Umberto I Hospital, Rome, Italy; 2 Dipartimento di Medicina Clinica e Chirurgia, Sezione di Endocrinologia, Università Federico II di Napoli, Naples, Italy; 3 Pituitary Unit, Department of Endocrinology and Metabolism, Fondazione Policlinico Universitario A. Gemelli, IRCCS, Rome, Italy; 4 Dipartimento di Medicina e Chirurgia Traslazionale, Università Cattolica del Sacro Cuore, Rome, Italy; 5 Endocrinology Unit, Department of Oncology and Medical Specialties, A.O. San Camillo-Forlanini, Rome, Italy; 6 Oxford Centre for Diabetes, Endocrinology and Metabolism, NIHR Oxford Biomedical Research Centre, University of Oxford, Churchill Hospital, Oxford, UK

## Abstract

**Objective:**

Registry data show that Cushing’s syndrome (CS) and adrenal insufficiency (AI) increase mortality rates associated with infectious diseases. Little information is available on susceptibility to milder forms of infections, especially those not requiring hospitalization. This study aimed to investigate infectious diseases in patients with glucocorticoid disorders through the development of a specific tool.

**Methods:**

We developed and administered the InfeCtions in pAtients with endocRinOpathies (ICARO) questionnaire, addressing infectious events over a 12-month observation period, to 1017 outpatients referred to 4 University Hospitals. The ICARO questionnaire showed good test–retest reliability. The odds of infection (OR (95% CI)) were estimated after adjustment for confounders and collated into the ICARO score, reflecting the frequency and duration of infections.

**Results:**

In total, 780 patients met the inclusion criteria: 43 with CS, 32 with adrenal incidentaloma and mild autonomous cortisol secretion (MACS), and 135 with AI, plus 570 controls. Compared to controls, CS was associated with higher odds of urinary tract infections (UTIs) (5.1 (2.3–9.9)), mycoses (4.4 (2.1–8.8)), and flu (2.9 (1.4–5.8)). Patients with adrenal incidentaloma and MACS also showed an increased risk of UTIs (3.7 (1.7–8.0)) and flu (3.2 (1.5–6.9)). Post-dexamethasone cortisol levels correlated with the ICARO score in patients with CS. AI was associated with higher odds of UTIs (2.5 (1.6–3.9)), mycoses (2.3 (1.4–3.8)), and gastrointestinal infections (2.2 (1.5–3.3)), independently of any glucocorticoid replacement dose.

**Conclusions:**

The ICARO tool revealed a high prevalence of self-reported infections in patients with glucocorticoid disorders. ICARO is the first of its kind questionnaire, which could be a valuable tool for monitoring infections in various clinical settings.

## Introduction

The advent of the novel coronavirus disease (COVID-19) in early 2020 changed the world. It led the scientific community, including endocrinologists (1, 2), to refocus on the importance of infectious diseases in endocrine patients. COVID-19 has shown that mild infections may be asymptomatic in some subjects but can turn into a life-threatening disease in more susceptible patients, in whom systemic complications determine the outcome (3, 4, 5, 6, 7). Endocrinologists thus know that endocrine patients at risk of complications from infectious diseases need special care but currently have no dedicated tools that can objectively estimate this risk.

Glucocorticoids (GCs) have a profound effect on host response, both stimulating and inhibiting the immune system and altering susceptibility to infections (8). Overt GC excess, known as Cushing’s syndrome (CS), has been associated with increased comorbidities, including cardiovascular disorders, diabetes, and immune suppression (9, 10, 11), while infectious diseases are considered one of the main causes of death in patients with active CS (12, 13, 14, 15). To date, only a few studies have reported the prevalence of infections, estimated as 21–51%, during the active phase of CS (9). However, these data were skewed by the inclusion of ectopic CS, a more severe condition with oncological confounders (16, 17, 18, 19) and, importantly, only refer to infections leading to hospitalization. It is therefore impossible to discern whether CS increases susceptibility to infections or increases their severity, leading to increased hospitalization. While possible autonomous cortisol secretion from adrenal incidentalomas (20), also called mild autonomous cortisol secretion (MACS) (21, 22), is related to an increased incidence of cardiovascular and metabolic complications (23, 24, 25), there is a lack of data on infectious diseases. Finally, there are no data on the commonest, mild infections, which endocrinologists often face in outpatient clinics.

On the other side of the coin, adrenal insufficiency (AI), or hypocortisolism, has also been associated with premature mortality, in some cases ascribed to adrenal crisis triggered by infectious diseases (26, 27, 28, 29, 30, 31). Registry data reveal that AI patients seem to use more antimicrobial agents and are more often hospitalized for infections than matched controls (32, 33). A recent trial revealed a higher cumulative incidence of different infections in AI patients in comparison with healthy controls (34). Again, less severe infections that do not require hospitalization or prescription drugs might be missed by public health or insurance registries – yet, these events are still capable of triggering complications and affecting quality of life (35, 36, 37).

To date, no cohort studies have investigated the prevalence, type, and severity of infections in patients with hypercortisolism (endogenous CS and MACS) or primary (PAI) and secondary (SAI) adrenal insufficiency. The aim of this study is to customize a new tool (a self-completed questionnaire) to assess susceptibility to infections in endocrine patients, focusing on patients with various degrees of GC excess or deficiency, in real-life outpatient settings.

## Subjects and methods

Between January 2018 and June 2019, we developed a questionnaire addressing infectious events in the previous 12 months and administered it to 1017 consecutive participants aged between 18 and 80 years attending the Endocrinology outpatient clinics at Sapienza University of Rome, Federico II University of Naples, Catholic University of Rome, or San Camillo-Forlanini Hospital of Rome. All patients were asked to complete a self-reported 30-item questionnaire on the type, frequency, duration, and treatment of any infectious diseases they had in the previous 12 months. All participants gave their informed verbal consent to completion of the questionnaire and underwent a full medical interview. The study was performed in accordance with the principles of the Declaration of Helsinki, and patient enrollment was authorized by the Sapienza University of Rome Ethics Committee (reference numbers 4945 and 7279). Questionnaires were administered confidentially, and the results were recorded under an anonymous alphanumeric code for each patient.

### The questionnaire

The questionnaire used (Supplementary data 1, see section on supplementary materials given at the end of this article) is a structured tool based on the German National Cohort (GNC) Questionnaire (38, 39) and the immune system assessment questionnaire (ISAQ) (40). Both questionnaires have been validated as self-administered tools with good test–retest reliability. Like them, our tool, named InfeCtions in pAtients with endocRinOpathies (ICARO), contains items on the type of infections occurring over the previous 12 months. These investigate upper respiratory tract infections (URTIs: colds and infections of the sinus, tonsils, middle ear, throat, and larynx), lower respiratory tract infections (LRTIs: bronchitis and pneumonia), gastrointestinal infections (‘stomach flu’) (GIIs), two clusters of skin and soft tissue infections (SSTIs-1: herpes labialis, genital herpes, warts, herpes zoster, and conjunctivitis and SSTIs-2: boils, abscesses, and styes), sexually transmitted infections (STIs), and urinary tract infections (UTIs). The questionnaire also investigated the frequency (none, one to two times; three to four times; five to six times, more than six times/year), duration (less than 1 week, 1–2 weeks, 2–3 weeks, more than 3 weeks), and treatment (no treatment, antibiotics, antivirals, antifungals) of these infections (38, 39). The ICARO score was developed as a quantitative measure to test whether the type or severity of the GC imbalance had any impact on the frequency and duration of infections. Frequency was weighted in accordance with the ISAQ validation (40): a negative response was weighted as 0.5, while ordinal frequency answers were reduced to binary items (i.e., one to two times and three times or more), with a weighting of 2 and 4, respectively. The duration of infections was also considered: the answer ‘less than 1 week’ was weighted as 0, while other answers were again reduced to binary items (i.e., 1–2 weeks and more than 2 weeks), with a weighting of 2 and 4, respectively. The arithmetic sum of the weights attributed to each item determined the total score (ICARO score). The range of possible scores is 4.5–68, with a higher score indicating a higher frequency and duration of infections. The observation time (12 months), derived from the GNC and ISAQ questionnaires, was chosen because it avoids biases due to seasonal variations in infection rates (38).

Compared to the GNC, we added two items addressing mycosis (MYC) and flu and omitted items not directly related to infections, as ICARO is designed for use in outpatient clinics, where the patient’s medical history (PMH) is available. In fact, ICARO is conceived to quantify susceptibility to infections independently of the underlying endocrine disorder, making it a single tool that can be offered to different types of endocrine patients; the inclusion of specific PMH questions would have limited this opportunity. We also added three items addressing hospital admission, absence from work, and vaccination (Supplementary data 1).

Demographic and clinical data were collected from patients’ medical records. The questionnaire was translated into Italian, and its translation was validated for comprehensibility. Although the questionnaire was self-reported, a clinician was always available for clarifications and to ensure all items were answered. Even though the GNC and ISAQ questionnaires have already been validated (38, 39, 40), the test–retest reliability of ICARO was also assessed randomly in a small group of patients (*n* = 20) who were asked to complete the questionnaire twice within 30 days (range: 8–28 days). Unweighted Cohen’s kappa coefficient was calculated for each question, with optimal concordance for all the questionnaire items (κ ≥ 0.85). Lastly, to assess the test–retest reliability of the questionnaire score, Cronbach’s alpha was calculated (α = 0.989) and interclass correlation (ICC) was evaluated with a two-way mixed model assuming absolute agreement. The ICC of single measurements was 0.978 (95% CI: 0.937–0.992). Overall, the test–retest reliability of the questionnaire was excellent. As an additional validating step, we had previously tested recall bias in a shorter version of the ICARO questionnaire used in the DREAM trial (34), in which recollected data from the questionnaire were paired with prospectively collected adverse events. There was a strong correlation between URTIs observed as adverse events and URTIs self-reported in the questionnaire (r = 0.986; *P* < 0.001), again supporting the questionnaire’s reliability and validation.

### Subjects

The groups of interest were subjects with endogenous hypercortisolism or hypocortisolism. The hypercortisolism group consisted of patients with endogenous CS and MACS. CS patients were subjects with pituitary ACTH-dependent or ACTH-independent hypercortisolism who had presented clinical signs and symptoms of CS for more than a year, and typical laboratory findings, according to guidelines (41, 42). They completed the questionnaire soon after the biochemical diagnosis of excess cortisol but before the commencement of any treatment. The MACS subgroup comprised patients with an incidental finding of adrenal adenoma, cortisol levels between 51 and 138 nmol/L after 1 mg overnight dexamethasone test (1 mg-DST), and a non-CS phenotype (20, 21). The questionnaire was administered to these patients soon after radiological and biochemical diagnosis. The hypocortisolism group comprised patients with PAI and SAI diagnosed according to guidelines and treated with GCs plus daily doses of fludrocortisone as needed (43). All patients were clinically stable, with no medication changes for their GC disorder in the 12 months before enrollment. All patients with hypocortisolism received an emergency kit and training in how to avoid adrenal crisis.

The control group consisted of consecutive endocrine patients attending the outpatient clinics for non-functioning adrenal adenomas, non-functioning pituitary adenomas without pituitary insufficiency, microprolactinomas, thyroid nodules, treated hypothyroidism, or well-controlled diabetes mellitus. The exclusion criteria were uncontrolled diabetes (Hba1c > 7.5%), ectopic CS, clinical or laboratory signs of significant hematologic, cerebral, cardiovascular, respiratory, hepatobiliary, pancreatic or kidney diseases, HIV, autoimmune polyendocrinopathy–candidiasis–ectodermal dystrophy, current malignant cancers (including thyroid cancer), severe psychiatric conditions, immunosuppressant or corticosteroid treatment for rheumatological, pneumological, or allergic conditions, pregnancy, or hyperthyroidism/hypothyroidism not adequately controlled at screening. Age, sex, smoking status, overweight/obesity, diabetes mellitus diagnosis, reproductive function, past medical history, disease duration, and medications were recorded for all subjects. In the hypocortisolism group, steroids were converted into daily hydrocortisone equivalent doses. Recruitment was set to a sample size of 1000, considered by Comfrey and Lee (1992) as ‘excellent’ for subsequent analyses (44).

### Statistical analysis

Population characteristics were summarized as counts and percentages for categorical variables and as means and standard deviations for continuous variables. Distribution of continuous variables was tested with the Shapiro–Wilk test; linearity was established by visual inspection of a scatterplot. Categorical variables were expressed as percentage and frequency; continuous variables were reported as mean and 95% CI or median and interquartile range (IQR) (25th–75th percentile). The association between categorical variables was determined using the chi-square test with continuity correction. For multiple ordinal variables, a* post hoc* analysis involving pairwise comparisons using multiple z-tests of two proportions with a Bonferroni correction was performed. Unpaired Student’s *t*-test and Mann–Whitney test were applied to compare groups, as appropriate. Multivariate logistic regression was used to estimate the odds of having any single type of infection, evaluated with adjustment for potential confounders (age, sex, diabetes, menopause/hypogonadism, obesity). Spearman’s correlation was performed to test the correlation between infection score and both GC dose and duration. Regression analysis was performed to estimate GC contributions to ICARO score variability. Because the questionnaire covered an observation time of 12 months, we considered dichotic comorbidities (with a duration of at least 1 year) as confounders, as opposed to variables that could change drastically over the year (such as body mass index, Hba1c, or arterial blood pressure). The sensitivity analysis was performed first by excluding patients with obesity or diabetes from the entire cohort, then by analyzing only the cohort of subjects with obesity or diabetes. In view of the multiple independent tests, *P*  values were adjusted using the false discovery rate (FDR) method (Benjamini and Hochberg) (45). FDR-corrected *P* < 0.05 was considered significant. Statistical analyses were conducted using the statistical software IBM SPSS statistics 20.

## Results

**Figure 1:**
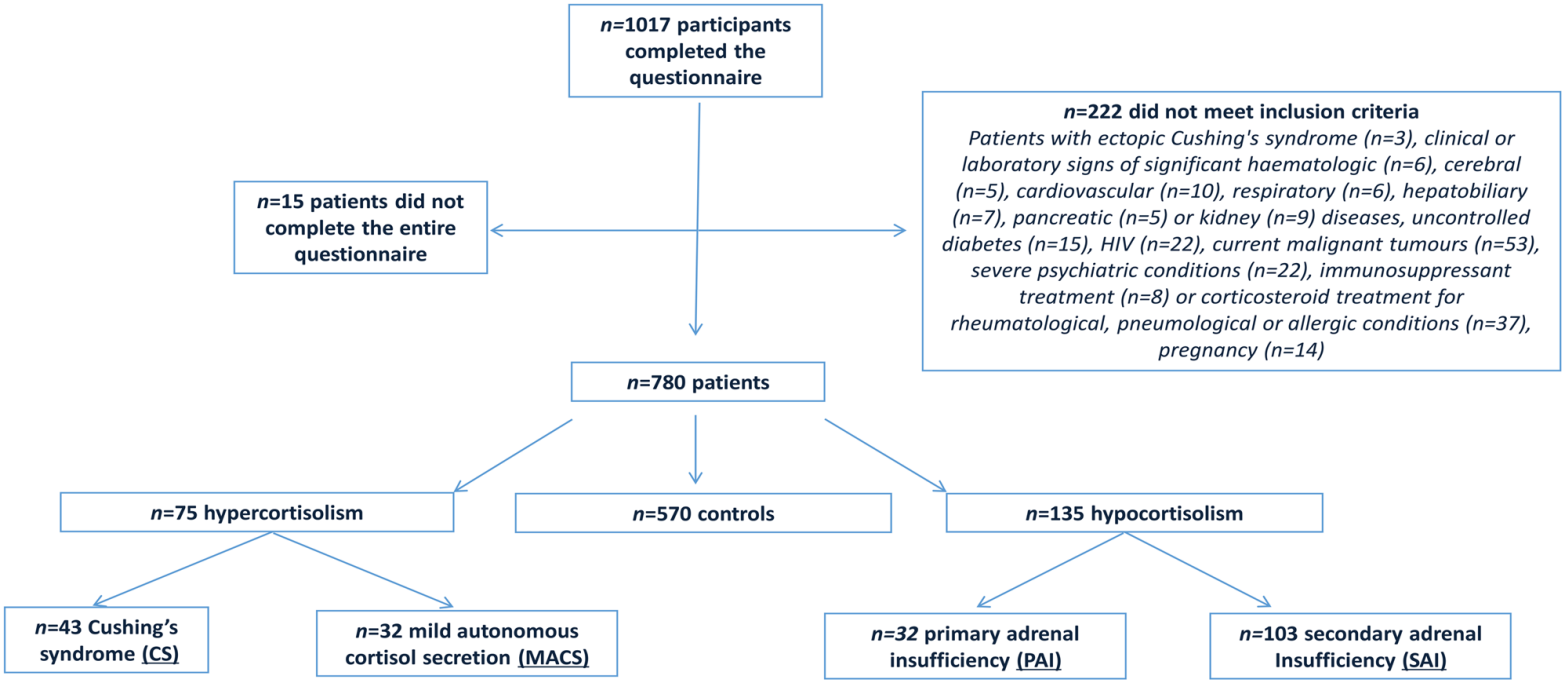
Study flow diagram.

Of the 1017 patients screened for study inclusion, 222 did not meet the inclusion criteria and 15 did not complete the entire questionnaire; 237 patients (23.3%) were therefore excluded from the analysis. The remaining 780 patients were enrolled (Fig. 1). Of these, 210 patients (26.9%) had GC disorders, and 570 (73.1%) comprised the control group. Among patients with GC disorders, 75 (35.7%) had a diagnosis of hypercortisolism (43 met the criteria for CS and 32 for MACS) and 135 (64.3%) of hypocortisolism (32 PAI and 103 SAI) (Fig. 1).

**Figure 2:**
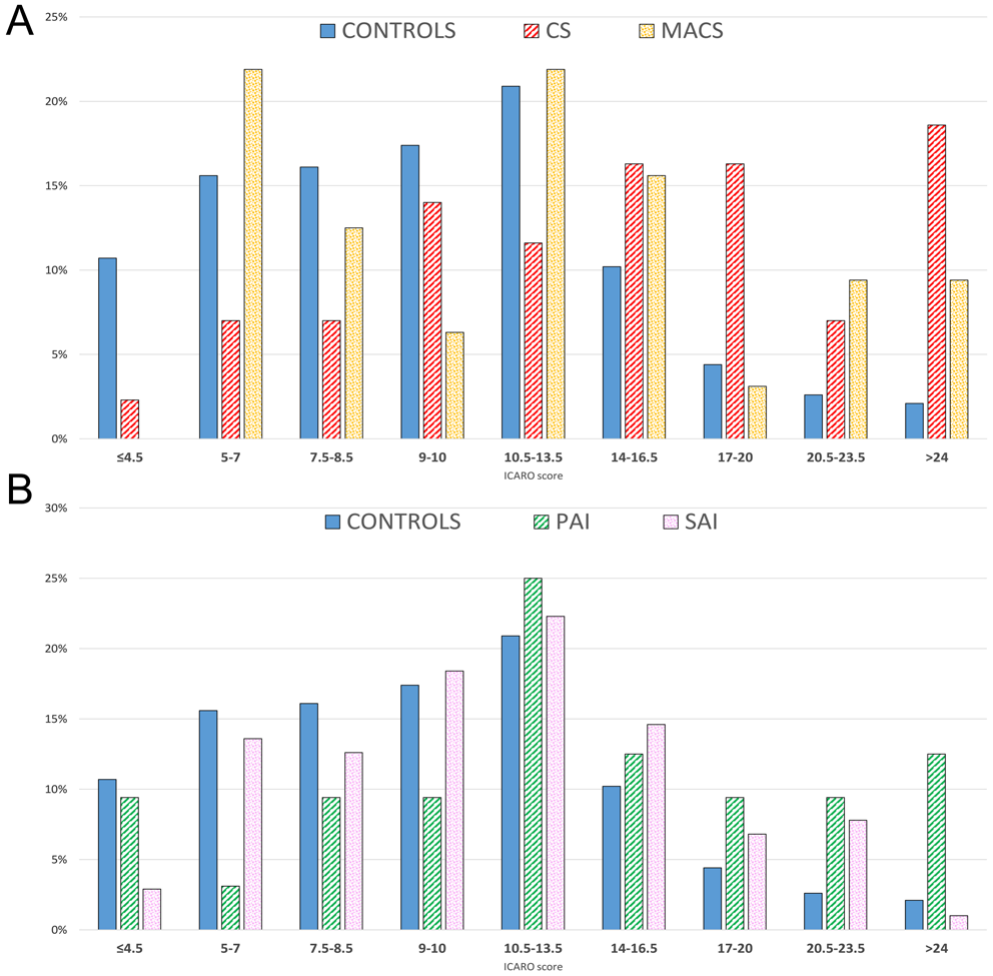
(A) ICARO score distribution in controls (blue), patients with Cushing’s syndrome (CS) (red lines), and patients with mild autonomous cortisol secretion (MACS) (orange dots). (B) ICARO score distribution in controls (blue), patients with primary adrenal insufficiency (PAI) (green lines), and patients with secondary adrenal insufficiency (SAI) (pink squares).

Tables 1 and 2 describe the clinical characteristics of the study population. The distribution of the total infection frequency and duration score (ICARO score), which measures the cumulative weighted incidence of different infections over 12 months, is reported in Fig. 2A and B. There were no differences in vaccination coverage, absence from work, or hospitalization due to infectious events between patients with CS, MACS, and hypocortisolism and the control group. Hospitalization was required for just 1% and antimicrobials for 48% of infections in patients with GC disorders. Statistically more antimicrobials were taken for URTIs (*P* < 0.001), MYC (*P* = 0.004), and UTIs (*P* = 0.029) lasting longer than 2 weeks than for those lasting less than 2 weeks.

**Table 1:** Clinical characteristics in controls and patients with Cushing’s syndrome (CS) and mild autonomous cortisol secretion (MACS). Categorical variables are expressed as frequency and percentages. Continuous variables are expressed as mean ± s.d.

	All subjects	Controls	CS	P*	MACS	P*
*n*	780	570	43		32	
Age (years)	49.9 ± 16	49.1 ± 16	46.2 ± 12	0.243	65 ± 8	<0.001
Females	424 (54%)	294 (52%)	37 (86%)	<0.001	24 (75%)	0.065
Diabetes	71 (9%)	38 (7%)	14 (33%)	<0.001	3 (9%)	1.000
Obesity	124 (16%)	78 (14%)	17 (39.5%)	<0.001	8 (25%)	0.514
Menopause/hypogonadism	382 (49%)	266 (47%)	20 (46.5%)	0.914	26 (81%)	0.002
Smokers	150 (19%)	99 (17%)	10 (23%)	0.443	8 (25%)	0.389

^*^P values for comparisons with controls.

**Table 2:** Clinical characteristics in controls, in patients with hypocortisolism, and in the subgroups of patients with primary adrenal insufficiency (PAI) and secondary adrenal insufficiency (SAI). Categorical variables are expressed as frequency and percentages. Continuous variables are expressed as mean and s.d.

	All subjects	Controls	Hypocortisolism	P*	PAI	P*	SAI	P*
*n*	780	570	135		32		103	
Age (years)	49.9 ± 16	49.1 ± 16	50.5 ± 15	0.460	50.3 ± 15	0.699	50.6 ± 15	0.421
Females	424 (54%)	294 (52%)	69 (51%)	0.998	17 (53%)	1.000	17 (16.5%)	0.829
Diabetes	71 (9%)	38 (7%)	16 (12%)	0.190	4 (12.5%)	0.366	12 (12%)	0.099
Obesity	124 (16%)	78 (14%)	21 (16%)	0.850	4 (12.5% )	1.000	17 (16.5%)	0.436
Menopause/hypogonadism	382 (49%)	266 (47%)	70 (52%)	0.968	15 (47%)	1.000	55 (53%)	0.207
Smokers	150 (19%)	99 (17%)	33 (24%)	0.263	6 (19%)	1.000	27 (27%)	0.188

^*^P values for comparisons with controls.

### Cushing’s syndrome

**Figure 3:**
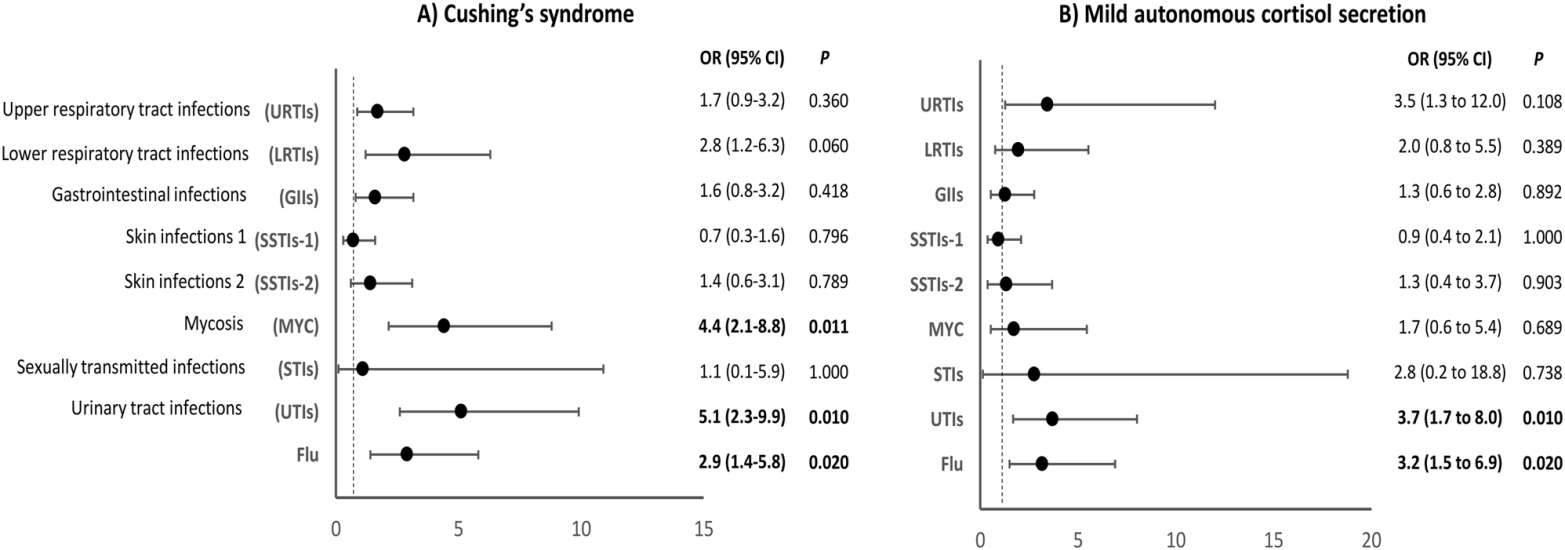
Odds of having an infectious disease in the 12 months prior to questionnaire administration in patients with Cushing’s syndrome (A) and mild autonomous cortisol secretion (B) compared with controls. The figure shows the summary of the odds ratios deriving from regression analysis performed for each infection and adjusted for age, sex, diabetes, obesity, and menopause/hypogonadism.

The prevalence of females, diabetes, and obesity was significantly higher in CS patients than controls (Table 1). The 43 CS patients (38 pituitary, 5 adrenal) had a median 1 mg-DST of 101 nmol/L (IQR 77–300) and typical clinical signs of CS. The multivariate logistic regression showed that these patients had higher adjusted odds of developing UTIs (OR: 5.1 (95% CI: 2.3–9.9); *P* = 0.010), MYC (OR: 4.4 (95% CI: 2.1–8.8); *P* = 0.011), and flu (OR: 2.9 (95% CI: 1.4–5.8); *P* = 0.020), compared to controls (Fig. 3). Patients with CS were also more likely to report at least one episode of MYC (42% vs 11%; *P* = 0.018), UTIs (53.5% vs 15%; *P* = 0.015), and flu (67% vs 42%; *P* = 0.021) in the timeframe covered by the questionnaire (Table 3).

**Table 3:** Prevalence and treatment of infectious diseases, hospitalization, vaccination, absence from work due to infectious disease, and ICARO score in controls and in patients with Cushing’s syndrome (CS) and mild autonomous cortisol secretion (MACS). Categorical variables are expressed as percentages and frequencies. Continuous variable is expressed as median (25th–75th percentile).

	Controls (*n* = 570)	CS (*n* = 43)	P*	MACS (*n* = 32)	P*	*P§*
Upper respiratory tract infections	75% (429/570)	81% (35/43)	0.738	87.5% (28/32)	0.150	0.693
Antimicrobials	36% (154/429)	40% (14/35)	0.742	46% (13/28)	0.925	0.819
Lower respiratory tract infections	11% (64/570)	23% (10/43)	0.086	25% (8/32)	0.150	0.861
Antimicrobials	73% (47/64)	80% (8/10)	0.892	87.5% (7/8)	0.985	0.769
Gastrointestinal tract infections	32.5% (185/570)	49% (21/43)	0.112	31% (10/32)	1.000	0.126
Antimicrobials	18% (33/185)	9.5% (2/21)	0.732	20% (2/10)	1.000	0.810
Skin infections 1	25% (142/570)	23% (10/43)	1.000	25% (8/32)	1.000	0.861
Antimicrobials	39% (55/142)	90% (9/10)	**0.019**	50% (4/8)	1.000	0.065
Skin infections 2	15% (88/570)	23% (10/43)	0.459	12.5% (4/32)	1.000	0.377
Antimicrobials	27% (24/88)	20% (2/10)	0.054	25% (1/4)	1.000	0.837
Mycosis	11% (63/570)	42% (18/43)	**0.018**	16% (5/32)	0.899	**0.029**
Antimicrobials	81% (51/63)	83% (15/18)	1.000	40% (2/5)	0.360	0.169
Sexually transmitted infections	2% (9/570)	2% (1/43)	0.951	3% (1/32)	1.000	1.000
Antimicrobials	56% (5/9)	100% (1/1)	0.752	0% (0/1)	1.000	1.000
Urinary tract infections	15% (86/570)	53.5% (23/43)	**0.015**	44% (14/32)	**0.013**	0.548
Antimicrobials	76% (65/86)	74% (17/23)	1.000	36% (5/14)	0.062	0.051
Flu	42% (240/570)	67% (29/43)	**0.021**	59% (19/32)	0.082	0.634
Hospitalization due to infectious disease	1% (5/570)	2% (1/43)	0.736	3% (1/32)	0.977	0.832
Vaccination	20% (112/570)	21% (9/43)	1.000	34% (11/32)	0.260	0.299
Absence from work due to infectious disease	10% (56/570)	12% (5/43)	0.948	3% (1/32)	0.757	0.362
ICARO score	9.5 (6–13)	16 (9–20.5)	**0.014**	11.25 (7.5–16.5)	**0.034**	**0.049**

After correction for multiple testing, *P* < 0.05 was considered statistically significant (bold).

^*^P values for comparisons with controls; ^§^*P* values for comparisons between CS and MACS.

Patients with CS also reported a longer duration of the infectious episode, specifically for GIIs (*P* = 0.037), SSTIs-2 (*P* = 0.034), and MYC (*P* = 0.016) (Supplementary Table 1), than the controls. They also took antimicrobials to treat SSTIs-1 significantly more often than controls (90% vs 39% respectively; *P* = 0.019) (Table 3). Finally, they had a higher ICARO score than controls (16 vs 9.5 respectively; *P* = 0.014).

Sensitivity analysis revealed that the median frequency of infection (score) was significantly higher in CS patients than controls, even after excluding patients with diabetes or obesity from the entire cohort (11.5 vs 9.5; *P* = 0.012). Similarly, a higher frequency of infection was also seen in this group when restricting the analysis to the subjects with diabetes or obesity (18.5 vs 9; *P* = 0.09).

### Mild autonomous cortisol secretion

MACS patients were significantly older than controls, with a higher prevalence of menopause/hypogonadism (Table 1). The 32 patients with MACS showed a median 1 mg-DST of 81.5 nmol/L (59-114). In this group, the multivariate logistic regression showed an increased adjusted odds of developing recurrent UTIs (OR: 3.7 (95% CI: 1.7–8.0); *P* = 0.010) and flu (OR: 3.2 (95% CI: 1.5–6.9); *P* = 0.020), compared to controls (Fig. 3). These patients were also more likely to report at least one episode of UTI (44% vs 15%; *P* = 0.013) (Table 3). They also had a higher ICARO score than the controls (11.25 vs 9.5; *P* = 0.034).

**Figure 4:**
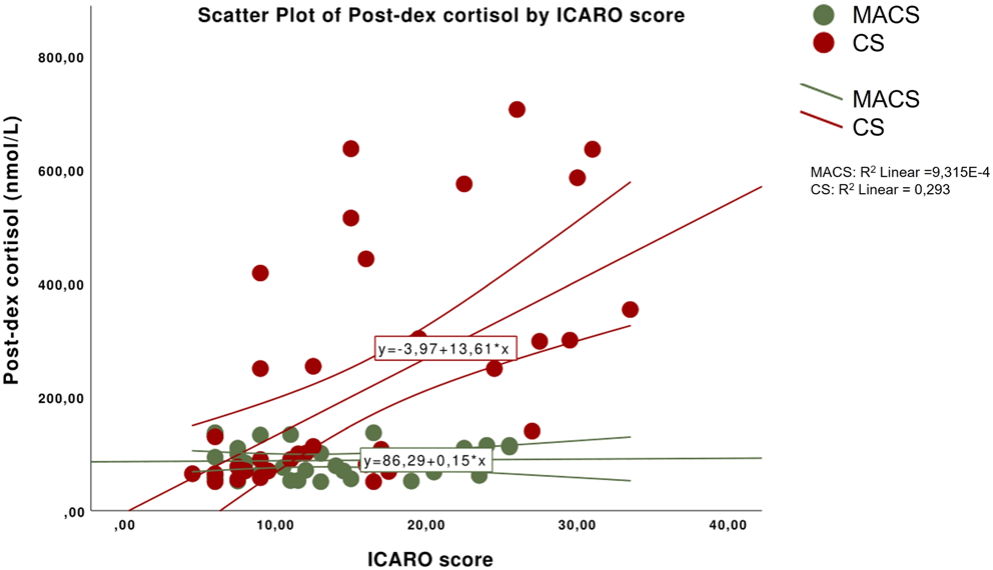
Correlation between post-dexamethasone (post-DEX) cortisol levels and ICARO score in patients with Cushing’s syndrome (CS) and mild autonomous cortisol secretion (MACS).

A linear regression analysis showed that the ICARO score was positively correlated with post-DST cortisol in patients with CS (r = 0.581, *P* < 0.001) but not in MACS patients (*P* = 0.958) (Fig. 4). In fact, the ICARO score was higher in CS (median: 16, IQR: 9–20.5) than MACS patients (median: 11.25, IQR: 7.5–16.5; *P* = 0.049). No statistical differences in prevalence, duration, and treatment of infections were found between CS and MACS patients, except for prevalence of MYC, which was higher in CS patients (42% vs 16% respectively; *P* = 0.029).

### Hypocortisolism: all subjects

**Figure 5:**
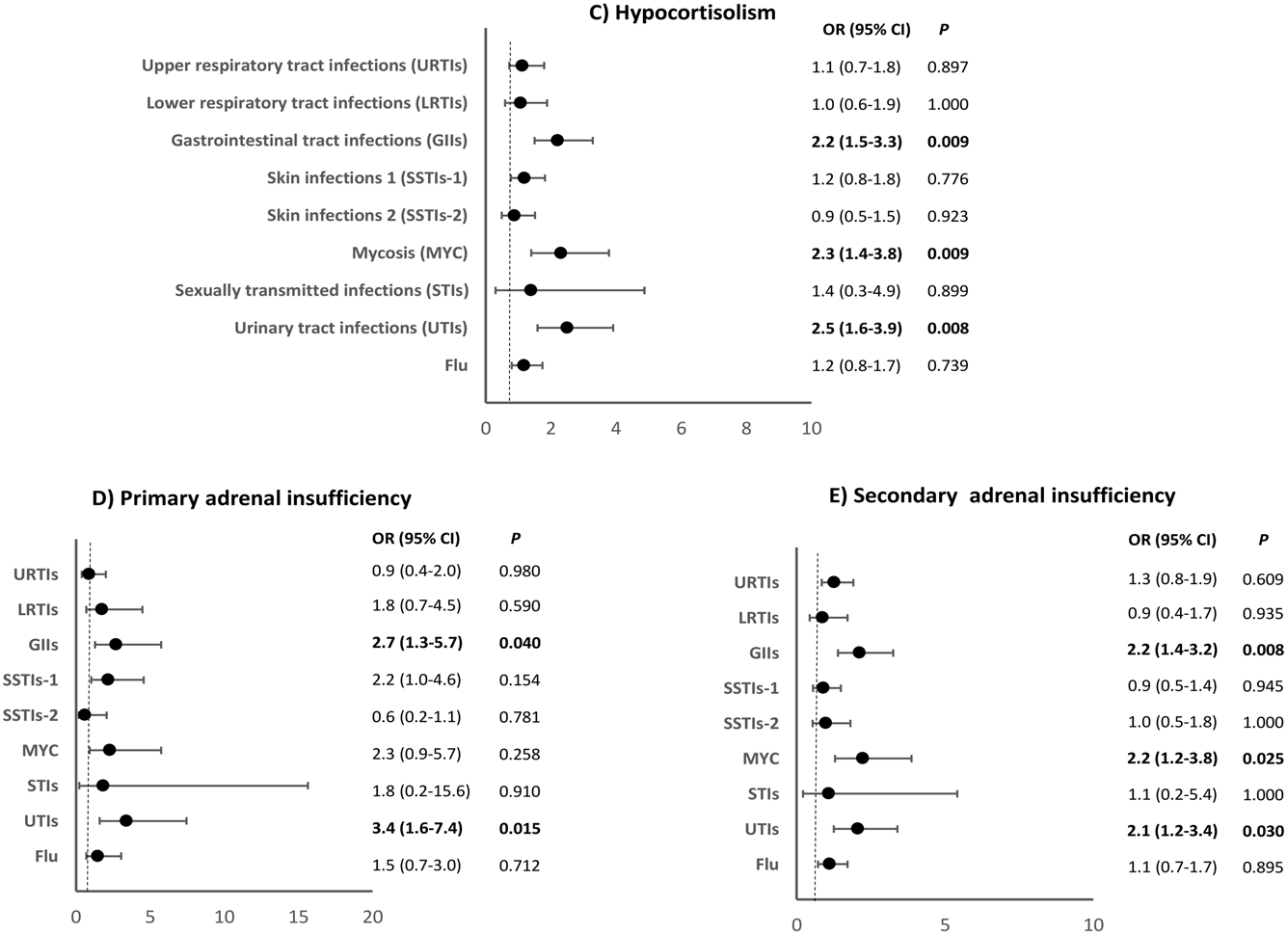
Odds of having an infectious disease in 12 months prior to questionnaire administration in patients with hypoadrenalism (C), primary adrenal insufficiency (D), and secondary adrenal insufficiency (E) compared with controls. The figure shows the summary of the odds ratios deriving from regression analysis performed for each infection and adjusted for age, sex, diabetes, obesity, and menopause/hypogonadism.

AI patients presented higher adjusted odds of developing frequent UTIs (OR: 2.5 (95% CI: 1.6–3.9); *P* < 0.008), MYC (OR: 2.3 (95% CI: 1.4–3.8); *P* = 0.009), and GIIs (OR: 2.2 (95% CI: 1.5–3.3); *P* = 0.009), compared with controls (Fig. 5). Patients with AI were more likely to experience at least one episode of GIIs (50% vs 32.5%; *P* = 0.013), MYC (22% vs 11%, *P* = 0.012), and UTIs (30% vs 15%; *P* = 0.030) (Table 4), as well as long-lasting LRTIs (*P* = 0.033) (Supplementary Table 2) than the controls. All AI patients were diagnosed at least 1 year before enrollment and had a stable replacement therapy with a median hydrocortisone equivalent dose of 20 mg/day (IQR: 20–30). There was no significant difference in the replacement dose between subgroups (20 mg/day, IQR: 20–25, in PAI patients and 25 mg/day, IQR: 15–30, in SAI patients, *P* = 0.968) or in the median GC therapy duration (4.5 years, IQR: 2–10, for PAI patients and 4 years, IQR: 2–9, for SAI patients, *P* = 0.707). A binomial logistic regression adjusted for these confounding factors excluded any major role of GC dose or duration of therapy as risk factors for GIIs, MYC, or UTIs.

**Table 4:** Prevalence and treatment of infectious diseases, hospitalization, vaccination, absence from work due to infectious disease, and ICARO score in controls, in patients with hypocortisolism, and in the subgroups of patients with primary adrenal insufficiency (PAI) and secondary adrenal insufficiency (SAI). Categorical variables are expressed as percentages and frequencies. Continuous variable is expressed as median (25th–75th percentile).

	Controls (*n* = 570)	Hypocortisolism (*n* = 135)	P*	PAI (*n* = 32)	P*	SAI (*n* = 103)	P*	*P§*
Upper respiratory tract infections	75% (429/570)	76% (103/135)	1.000	72% (23/32)	1.000	78% (80/103)	0.907	0.663
Antimicrobials	36% (154/429)	37% (38/103)	1.000	48% (11/23)	0.739	30% (24/80)	0.900	0.112
Lower respiratory tract infections	11% (64/570)	13% (17/135)	0.992	19% (6/32)	0.714	11% (11/103)	1.000	0.327
Antimicrobials	73% (47/64)	76.5% (13/17)	1.000	83% (5/6)	1.000	73% (8/11)	1.000	0.622
Gastrointestinal tract infections	32.5% (185/570)	50% (68/135)	**0.013**	53% (17/32)	0.110	49.5% (51/103)	**0.015**	0.721
Antimicrobials	18% (33/185)	13% (9/68)	0.842	23.5% (4/17)	1.000	10% (5/51)	0.405	0.302
Skin infections 1	25% (142/570)	28% (38/135)	0.848	41% (13/32)	0.262	24% (25/103)	0.984	0.116
Antimicrobials	39% (55/142)	47% (18/38)	0.797	38.5% (5/13)	1.000	40% (10/25)	0.835	0.927
Skin infections 2	15% (88/570)	13% (18/135)	0.892	9% (3/32)	0.841	15% (15/103)	1.000	0.648
Antimicrobials	27% (24/88)	44% (8/18)	0.598	33% (1/3)	1.000	47% (7/15)	0.387	0.671
Mycosis	11% (63/570)	22% (30/135)	**0.012**	22% (7/32)	0.359	22% (23/103)	**0.020**	0.957
Antimicrobials	81% (51/63)	83% (25/30)	1.000	86% (6/7)	1.000	83% (19/23)	1.000	0.847
Sexually transmitted infections	2% (9/570)	2% (3/135)	1.000	3% (1/32)	1.000	2% (2/103)	1.000	0.692
Antimicrobials	56% (5/9)	33% (1/3)	1.000	0% (0/1)	1.000	50% (1/2)	1.000	1.000
Urinary tract infections	15% (86/570)	30% (41/135)	**0.030**	38% (12/32)	**0.015**	28% (29/103)	**0.033**	0.433
Antimicrobials	76% (65/86)	61% (25/41)	0.393	33% (4/12)	0.062	69% (20/29)	1.000	0.079
Flu	42% (240/570)	45% (61/135)	0.515	50% (16/32)	0.379	44% (45/103)	0.825	0.531
Hospitalization due to infectious disease	1% (5/570)	1.5% (2/135)	1.000	0% (0/32)	1.000	2% (2/103)	0.906	0.427
Vaccination	20% (112/570)	21.5% (29/135)	0.960	31.% (10/32)	0.453	19% (19/103)	1.000	0.196
Absence from work due to infectious disease	10% (56/570)	13% (17/135)	0.787	6% (2/32)	0.950	15% (15/103)	0.461	0.351
ICARO score	9.5 (6–13)	11.5 (8–15)	**0.038**	11.75 (9.5–19.75)	**0.021**	11 (7.5–15)	0.067	0.123

After correction for multiple testing, *P* < 0.05 was considered a statistically significant difference (bold).

^*^P values for comparisons with controls; ^§^*P* values for comparisons between PAI and SAI.

Patients with AI had a significantly higher ICARO score than the controls (11.5 vs 9.5; *P* = 0.038), even after controlling for daily GC dose and duration and excluding subjects with obesity and diabetes (11.5 vs 9.5; *P* = 0.035). No differences in prevalence, duration, or ICARO score were found between primary and secondary AI.

### Primary adrenal insufficiency

A subgroup analysis was performed in patients with hypocortisolism. The PAI subgroup showed higher adjusted odds of developing recurrent GIIs (OR: 2.7 (95% CI: 1.3–5.7); *P* = 0.040) and UTIs (OR: 3.4 (95% CI: 1.6–7.4);* P* = 0.015), compared to controls (Fig. 5) and were more likely to report at least one episode of UTI (38% vs 15%; *P* = 0.015) (Table 4). The average URTI duration was higher in PAI patients compared to controls (*P* = 0.036) (Supplementary Table 2).

### Secondary adrenal insufficiency

These patients had increased adjusted odds of developing GIIs (OR: 2.2 (95% CI: 1.4–3.2); *P* = 0.008), MYC (OR: 2.2 (95% CI: 1.2–3.8); *P* = 0.025), and UTIs (OR: 2.1 (95% CI:, 1.2–3.4); *P* = 0.030) compared to controls (Fig. 5). They were also more likely to experience at least one episode of GIIs (49.5% vs 32.5%; *P* = 0.015), MYC (22% vs 11%; *P* = 0.020), and UTIs (28% vs 15%; *P* = 0.033) (Table 4).

## Discussion

This study reveals that the disruption of endogenous GC levels, irrespective of the underlying cause, is associated with increased susceptibility to infections, as documented by higher frequencies and longer durations of self-reported episodes measured with a new tool, ICARO, specifically developed to assess infection risk in endocrine patients.

Although more than 50 years have passed since bacterial infections were first recognized as the leading cause of death in patients with CS (46), very few studies have addressed the epidemiology of infections in these patients (16, 17, 18, 19). Retrospective studies report infections mostly due to opportunistic pathogens but are biased by the inclusion of ectopic CS (16, 17, 18, 19). Others reported infections are frequent comorbidities in up to 21% of CD patients but without describing the type and severity (47). A recent retrospective study on CD confirmed five-fold increase in mortality due to infections (14). The mechanisms through which GC excess affects the innate and adaptive immune system were recently reviewed (8, 9), but no studies have investigated the risk of common, mild, and/or recurrent infections in CS, probably because they evade surveillance strategies (hospital record/insurance registries) and there were no tools previously capable of measuring their prevalence. Our study fills this gap and adds important information about the prevalence, odds, and duration of the commonest infections in a cohort that included 75 patients with hypercortisolism. In these patients, we measured a 2- to 5-fold increase in the odds for MYC, UTIs, and flu, compared to controls. This held true even after adjusting for age, sex, hypogonadism/menopausal state, obesity, and diabetes, suggesting that the infection risk is independent of the commonest confounding factors. We also revealed that GIIs, SSTIs-2, and MYC can last longer in these patients. Moreover, patients with CS took antimicrobials to treat skin infections significantly more often than controls, suggesting a higher aggressiveness of this type of infection. Awareness of the type, characteristics, and duration of infections could have clinical implications, considering the atypical presentations of infectious disease in patients with CS, in whom hypercortisolism can mitigate clinical symptoms, leading to possible referral delays (8). Interestingly, in CS patients, 1 mg-DST values are positively correlated with the ICARO score, suggesting a close relationship between the degree of hypercortisolism and the severity of infections.

Our analysis also included patients with adrenal incidentalomas and MACS (21). Between 30 and 50% of patients with adrenal incidentalomas have biochemical evidence of possible, low-grade autonomous cortisol release (20), associated with a worsened cardiovascular risk (hypertension, diabetes, and obesity), a higher prevalence of cardiovascular events, greater bone loss, and a higher mortality rate (21, 24, 48, 49, 50). However, very little attention has been paid to the infection risk in patients with MACS. To the best of our knowledge, only one retrospective study has investigated patients with adrenal incidentaloma and MACS, reporting higher infection-related mortality than in controls, but no data on prevalence and risk of infections were reported (51). Our study is the first to show that even a low‐grade cortisol excess results in a significant increase in some (UTIs and flu), but not all, infections.

Our data, as expected, suggest different phenotypes in hypercortisolemic patients: the increased odds for MYC in CS patients is not present in patients with MACS, while the increased frequency of UTIs and flu is observed in both groups. Furthermore, the positive correlation between post-DST cortisol and ICARO score was confirmed only for patients with CS, and not in patients with MACS.

Other than hypercortisolism, our results show a greater prevalence and odds of contracting GIIs, MYC, and UTIs in hypocortisolemic patients, who also experienced a longer duration of LRTIs.

Large retrospective studies have demonstrated that mortality is increased in chronic AI and is strongly correlated with infectious disease (26, 27, 28, 31, 52, 53). According to Swedish registry data, one-third of patients with PAI and concomitant diabetes suffered from infections requiring hospital admission (27). A recent British primary care retrospective database reported an increased risk of infections (LRTIs, UTIs, and GIIs) and an increased number of antimicrobial prescriptions in patients with PAI compared to population-based matched controls (54). In patients with AI, prompt treatment of infectious diseases is essential to minimize hospitalization and reduce adrenal crisis (37). It is also important to emphasize that infections are the most common precipitating factor for adrenal crisis, especially gastroenteritis and bronchopulmonary and urinary tract infections (37, 55, 56). The differences in the low incidence of hospitalization found in our study compared with the high incidence reported in previous data might be explained by the different observation periods: 12 months in this study contraposed with several years in the retrospective studies (27, 32). Our whole cohort of patients were also correctly informed about the risk of adrenal crisis, received an emergency kit, and were trained in how to avoid adrenal crisis; this could also help explain their low level of hospitalization.

In this regard, it is still unclear whether the higher risk of infections is correlated with GC replacement therapy doses or regimens in any way. We recently showed that patients with AI exhibit a selective immune impairment, mainly of innate immunity, which can be partially restored by a more circadian rhythm of GC administration (34, 57, 58, 59). This immune impairment was further linked to circadian gene disruption in AI patients (60). Our study is the first to show that patients with AI have a high risk of infectious disease, regardless of their GC total daily dose. However, in our cohort, the median hydrocortisone equivalent dose was 20 mg/day, and patients were not overtreated. Further studies are needed to confirm these findings and to better clarify the contribution of a tailored circadian replacement treatment in AI.

Our study has some limitations. First, caution should be exercised in the interpretation of our results, given that the data were self-reported and recall bias should be considered, with some infections possibly being counted by the patient more than once. However, in a shorter version of the ICARO questionnaire used previously in the DREAM trial, we established that recall bias was minimal (34). Moreover, the questionnaire was derived from validated questionnaires with a similar time frame and administered in the outpatient clinics of University Hospitals to participants who are used to remembering and answering questions about their conditions and symptoms.

Another limitation is the baseline differences in some demographic characteristics of the hypercortisolism group. This is a common occurrence in observational studies and is a consequence of the nature of this disease. As expected, hypercortisolism was more prevalent in women, and patients more frequently had diabetes and obesity than controls (9). We also found that most patients with MACS were older, and this is also consistent with previous large studies (49, 51, 61). To fix all these biases, we adjusted the odds ratio for the main confounders predisposing to infections (age, sex, diabetes, menopause/hypogonadism, obesity) and performed a sensitivity analysis excluding patients with obesity and diabetes. In addition, vaccination coverage, which could have been a confounding factor for infection prevalence, was similar for all groups.

In view of the scant original data on this topic, we believe that this study offers several benefits. It is the first objective tool to quantify the burden of infectious disease in endocrine patients. Patient-reported outcome measures (PROMs) like self-reported questionnaires are gaining momentum, especially in patients living with rare disorders, and their routine use in clinical practice has been shown to improve patient satisfaction with their care, management, and quality of life (62). The aforementioned ISAQ was recently tested in patients with PAI, with unsatisfactory results: this may have been due to the nature of the questionnaire, which was originally designed for severely immunocompromised patients (63). In contrast, the ICARO questionnaire is derived from both the GNC questionnaire and ISAQ and has been specifically adapted for outpatients. It could thus be a useful tool for future studies aiming to explore the relationships between infection type and severity, hormone levels, and treatment of endocrine disorders, proving capable of investigating the epidemiology of the most common infections experienced by patients, including infectious episodes that do not lead to hospitalization or death. These could easily be missed by registry/insurance sources, as patients are less likely to seek medical advice for trivial infections (UTIs, SSTIs, or flu), which, however, could still potentially trigger a more dangerous condition and impair quality of life. Further studies using the ICARO tool might even find a cut-off score that could offer additional information about the severity of infectious comorbidity and thus help in therapeutic decision-making.

In conclusion, the self-reported ICARO questionnaire may be a simple, cost-effective new PROM that is able to: (a) identify patients at a higher risk of infection and evaluate the effect of specific treatments on their susceptibility to infection; (b) provide both clinicians and patients with important information on vulnerability to infection; and (c) improve patients’ care and quality of life.

## Supplementary Material

Supplementary Material

Supplementary Table 1. Duration of infectious diseases in previous 12 months in controls and in patients with Cushing’s Syndrome (CS) and mild autonomous cortisol secretion (MACS). Categorical variables are expressed as percentages and frequencies. p* values for comparisons with controls. p§ values for comparisons between CS and MACS. After correction for multiple testing, p < 0.05 was considered as statistically significant (bold). a,b,c,d,e,f,g,h,i: significantly different duration compared with controls at post hoc analysis using multiple z-tests with Bonferroni correction.

Supplementary Table 2. Duration of infectious diseases occurring in previous 12 months in patients with hypocortisolism and in the subgroups of patients with primary adrenal insufficiency (PAI) and secondary adrenal insufficiency (SAI). Categorical variables are expressed as percentages and frequencies. p* values for comparisons with controls. p§ values for comparisons between PAI and SAI. After correction for multiple testing a p < 0.05 was considered as significant statistically different (bold). a,b.c significantly different duration compared with controls at post hoc analysis using multiple z-tests with Bonferroni correction.

## Declaration of interest

The authors declare that there is no conflict of interest that could be perceived as prejudicing the impartiality of the research reported.

## Funding

This work was partially funded by the CHRONOIMAGE project (PRIN 2017HRTZYA) by MIUR and by the PRecisiOn Medicine to Target Frailty of Endocrine-metabolic Origin (PROMETEO) project (NET-2018-12365454) by Ministry of Health http://dx.doi.org/10.13039/100009647.
