# Supplementary material for: Susceptibility and characteristics of infections in patients with glucocorticoid excess or insufficiency: the ICARO tool
Source: Eur J Endocrinol. 2022 Sep 14;187(5):719–31. doi: 10.1530/EJE-22-0454 (PMC9641788; doi:10.1530/EJE-22-0454)
Supplement: Supplementary Table 1. Duration of infectious diseases in previous 12 months in controls and in patients with Cushing’s Syndrome (CS) and mild autonomous cortisol secretion (MACS). Categorical variables are expressed as percentages and frequencies. p* values for comparisons with controls. p§ values  [file supplementary_table_1.pdf]

Supplementary Table 1. Duration of infectious diseases in previous 12 months in controls and in patients with Cushing's Syndrome (CS) and mild autonomous cortisol secretion (MACS). Categorical variables are expressed as percentages and frequencies. p* values for comparisons with controls. $p^{\S}$ values for comparisons between CS and MACS. After correction for multiple testing, $p < 0.05$ was considered as statistically significant (bold). [a,b,c,d,e,f,g,h,i]: significantly different duration compared with controls at post hoc analysis using multiple z-tests with Bonferroni correction.

	**Controls (n=570)**	**CS (N=43)**	***p****	**MACS (n=32)**	***p****	***p§***
**Upper Respiratory tract infections (URTIs) - duration**						
less than 1 week	65% (279/429)	66% (23/35)	0.903	54% (15/28)	0.742	0.516
from 1 to 2 weeks	28% (122/429)	26% (9/35)		39% (11/28)		
from 2 to 3 weeks	4% (19/429)	9% (3/35)		7% (2/28)		
more than 3 weeks	2% (9/429)	0% (0)		0% (0)		
**Lower respiratory tract infections (LRTIs) - duration**						
less than 1 week	30% (19/64)	10% (1/10)	0.060	12.5% (1/8)	0.742	0.409
from 1 to 2 weeks	55% (35/64)	60% (6/10)		87.5% (7/8)		
from 2 to 3 weeks	6% (3/64)	20% (2/10)		0% (0)		
more than 3 weeks	9% (6/64)	10% (1/10)		0% (0)		
**Gastrointestinal tract infections (GIIs) - duration**						
less than 1 week	88% (163/185)[a]	57% (12/21)[a]	**0.037**	65% (11/17)	0.114	0.946
from 1 to 2 weeks	11% (20/185)[b]	29% (6/21)[b]		29% (5/17)		
from 2 to 3 weeks	0.5% (1/185)	0% (0)		0% (0)		
more than 3 weeks	0.5% (1/185)[c]	14% (3/21)[c]		6% (1/17)		
**Skin infections 1 (SSTIs-1) - duration**						
less than 1 week	56% (79/142)	50% (5/10)	0.790	75% (6/8)	0.901	0.407
from 1 to 2 weeks	30% (42/142)	20% (2/10)		25% (2/8)		
from 2 to 3 weeks	6% (9/142)	20% (2/10)		0% (0)		
more than 3 weeks	8% (12/142)	10% (1/10)		0% (0)		
**Skin infections 2 (SSTIs-2) - duration**						
less than 1 week	56% (49/88)[d]	20% (2/10)[d]	**0.034**	50% (2/4)	0.290	0.540
from 1 to 2 weeks	33% (29/88)	30% (3/10)		25% (1/4)		
from 2 to 3 weeks	2% (2/88)[e]	20% (2/10)[e]		25% (1/4)		
more than 3 weeks	9% (8/88)[f]	30% (3/10)[f]		0% (0)		
**Mycosis (MYC) - duration**						
less than 1 week	33% (21/63)[g]	33% (6/18)[g]	**0.016**	60% (3/5)	0.894	0.062
from 1 to 2 weeks	48% (30/63)[h]	6% (1/18)[h]		40% (2/5)		
from 2 to 3 weeks	5% (3/63)[i]	33% (6/18)[i]		0% (0)		
more than 3 weeks	14% (9/63)	28% (5/18)		0% (0)		
**Sexually transmitted infections (STIs) - duration**						
less than 1 week	22% (2/9)	100% (1/1)	0.651	100% (1/1)	0.644	0.143
from 1 to 2 weeks	67% (6/9)	0% (0)		0% (0)		
from 2 to 3 weeks	0% (0)	0% (0)		0% (0)		
more than 3 weeks	11% (1/9)	0% (0)		0% (0)		
**Urinary tract infections (UTIs) - duration**						
less than 1 week	70% (59/86)	56.5% (13/23)	0.802	64% (9/14)	0.758	0.843
from 1 to 2 weeks	24% (21/86)	26% (6/23)		14% (2/14)		
from 2 to 3 weeks	2% (2/86)	4% (1/23)		7% (1/14)		
More than 3 weeks	5% (4/86)	13% (3/23)		14% (2/14)		
