# Supplementary material for: Susceptibility and characteristics of infections in patients with glucocorticoid excess or insufficiency: the ICARO tool
Source: Eur J Endocrinol. 2022 Sep 14;187(5):719–31. doi: 10.1530/EJE-22-0454 (PMC9641788; doi:10.1530/EJE-22-0454)
Supplement: Supplementary Table 2. Duration of infectious diseases occurring in previous 12 months in patients with hypocortisolism and in the subgroups of patients with primary adrenal insufficiency (PAI) and secondary adrenal insufficiency (SAI). Categorical variables are expressed as percentages and frequenc [file supplementary_table_2.pdf]

Supplementary Table 2. Duration of infectious diseases occurring in previous 12 months in patients with hypocortisolism and in the subgroups of patients with primary adrenal insufficiency (PAI) and secondary adrenal insufficiency (SAI). Categorical variables are expressed as percentages and frequencies. *p** values for comparisons with controls. $p^{\S}$ values for comparisons between PAI and SAI. After correction for multiple testing a $p < 0.05$ was considered as significant statistically different (bold). [a,b,c] significantly different duration compared with controls at post hoc analysis using multiple z-tests with Bonferroni correction.

	**Controls** (n=570)	**Hypocortisolism** (n=135)	*p**	**PAI** (n=32)	*p**	**SAI** (n=103)	*p**	$p^{\S}$
**Upper respiratory tract infections (URTIs) - duration**								
less than 1 week	65% (279/429)[a]	53% (55/103)		35% (8/23)[a]		59% (47/80)		
from 1 to 2 weeks	28% (122)	39% (40)	0.459	45.5% (10)	**0.036**	37.5% (30)	0.748	0.058
from 2 to 3 weeks	4% (19)	5% (5)		13% (3)		2.5% (2)		
more than 3 weeks	2% (9) [b]	3% (3)		8.5% (2)[b]		1% (1)		
**Lower respiratory tract infections (LRTIs) - duration**								
less than 1 week	30% (19/64)	7% (1)		33% (2/6)		9% (1/11)		
from 1 to 2 weeks	55% (35)	33% (5)	**0.033**	17% (1)	0.291	36% (4)	0.143	0.611
from 2 to 3 weeks	6% (3)[c]	35% (6) [c]		33% (2)		36% (4)		
more than 3 weeks	9% (6)	18% (3)		17% (1)		18% (2)		
**Gastrointestinal tract infections (GIIs)- duration**								
less than 1 week	88% (163/185)	75% (51/68)		65% (11/17)		65% (11/17)		
from 1 to 2 weeks	11% (20)	23.5% (13)	0.200	29% (5)	0.083	29% (5)	0.736	0.161
from 2 to 3 weeks	0.5% (1)	0% (0)		0% (0)		0% (0)		
more than 3 weeks	0.5% (1)	1.5% (1)		6% (1)		6% (1)		
**Skin infections 1 (SSTIs-1) - duration**								
less than 1 week	56% (79/142)	50% (19/38)		38.5% (5/13)		9% (1/11)		
from 1 to 2 weeks	30% (42)	32% (12)	1.000	23% (3)	0.469	36% (4)	0.141	0.098
from 2 to 3 weeks	6% (9)	5% (2)		15% (2)		36% (4)		
more than 3 weeks	8% (12)	13% (5)		23% (3)		18% (0)		
**Skin infections 2 (SSTIs-2) - duration**								
less than 1 week	56% (49/88)	67% (12/18)		100% (3/3)		56% (14/25)		
from 1 to 2 weeks	33% (29)	28% (5)	0.795	0% (0)	0.844	36% (9)	0.879	0.407
from 2 to 3 weeks	2% (2)	5% (1)		0% (0)		0% (1)		
more than 3 weeks	9% (8)	0% (0)		0% (0)		8% (2)		
**Mycosis (MYC) - duration**								
less than 1 week	33% (21/63)	33% (10/30)		43% (3/7)		30% (7/23)		
from 1 to 2 weeks	48% (30)	10% (3)	0.076	29% (2)	0.893	4% (1)	0.228	0.052
from 2 to 3 weeks	5% (3)	40% (12)		0% (0)		52% (12)		
more than 3 weeks	14% (9)	17% (5)		29% (2)		13% (3)		
**Sexually transmitted infections (STIs) - duration**								
less than 1 week	22% (2/9)	33% (1/3)		0% (0/1)		50% (1/2)		
from 1 to 2 weeks	67% (6)	33% (1)	0.547	0% (0)	0.347	50% (1)	0.897	0.223
from 2 to 3 weeks	0% (0)	33% (1)		0% (0)		0% (0)		
more than 3 weeks	11% (1)	33% (1)		100% (1)		0% (0)		
**Urinary tract infections (UTIs) duration**								
less than 1 week	70% (59/86)	73% (30/41)		75% (9/12)		72% (21/29)		
from 1 to 2 weeks	24% (21)	22% (9)	1.000	25% (3)	1.000	21% (6)	1.000	0.824
from 2 to 3 weeks	2% (2)	2.5% (1)		20% (0)		3% (1)		
more than 3 weeks	5% (4)	2.5% (1)		0% (0)		3% (1)		
